# Activity behaviours in British 6-year-olds: cross-sectional associations and longitudinal change during the school transition

**DOI:** 10.1123/jpah.2021-0718

**Published:** 2022-07-21

**Authors:** Kathryn R. Hesketh, Soren Brage, Hazel M. Inskip, Sarah R. Crozier, Keith M. Godfrey, Nicholas C. Harvey, Cyrus Cooper, Esther M.F. Van Sluijs

**Affiliations:** 1 MRC Epidemiology Unit, Institute of Metabolic Science, Cambridge Biomedical Campus, Cambridge, UK; 2 MRC Lifecourse Epidemiology Centre, University of Southampton, Southampton General Hospital, Southampton, UK; 3 NIHR Southampton Biomedical Research Centre, University of Southampton and University Hospital Southampton NHS Foundation Trust, Southampton, UK; 4 NIHR Applied Research Collaboration Wessex, Southampton Science Park, Southampton, UK; 5 -NIHR Biomedical Research Centre, University of Oxford, Oxford, UK

**Keywords:** Physical activity, sedentary, cohort, accelerometer, change

## Abstract

**Background:**

To explore activity behaviours at school entry, we describe temporal/demographic associations with accelerometer-measured physical activity in a population-based sample of British 6-year-olds, and examine change from age 4-6.

**Methods:**

712 6-year-olds (308 at both ages) wore Actiheart accelerometers for ≥3 (mean 6.0) days. We derived minutes/day sedentary (<20cpm) and moderate-to-vigorous physical activity (MVPA, ≥460cpm), also segmented across mornings (06:00-09:00), school (09:00-15:00) and evenings (15:00-23:00). Using mixed-effects linear regression, we analyzed associations between temporal/demographic factors and children’s activity intensities at age 6, and change between ages 4-6.

**Results:**

6-year-old children engaged in mean(SD) MVPA: 64.9(25.7) minutes/day (53% met UK guidelines). Girls did less MVPA than boys, particularly during school hours. Children were less active at weekends (vs. weekdays) and more active on spring/summer evenings (vs. winter). Longitudinally, 6-year-old children did less LPA (-44.7 [95%C.I.:-49.9,-39.6] minutes/day) but were more sedentary (30.0 [24.5,35.5]) and engaged in greater MVPA (7.6 [5.6,9.7]) compared with when aged 4.

**Conclusion:**

Half of 6-year-old children met current activity guidelines; MVPA levels were lower in girls and at weekends. UK children became more sedentary but did more MVPA as they entered formal schooling. PA promotion efforts should capitalise on these changes in MVPA, to maintain positive habits.

## Introduction

Physical activity is beneficial for children’s physical and mental health,^1^ yet activity levels decrease across childhood and adolescence,^2^ and into adulthood.^3^ Ensuring young children meet physical activity guidelines to establish a good baseline level of physical activity is therefore important.

A child’s transition to formal schooling (the September before they turn 5 years old in the UK) may be a key time when behaviours and habits change and are consolidated. This transition to school coincides with changing physical activity guidelines, and therefore differing ideas of what constitutes ‘sufficiently active’.^4^ Current UK physical activity guidelines recommend that children under 5 years engage in 180 minutes of activity at any intensity, including 60 minutes MVPA in children aged 3-4 years; from age 5 (to 18) years, an average of 60 minutes of moderate to vigorous physical activity (MVPA) per day across the week is recommended.^4^ For all children it is also recommended that extended periods of sedentary time (e.g. sitting) be minimised.^4^ UK activity guidelines are consistent with many recommendations worldwide.^5^

Self-report measures of physical activity and sedentary behaviour in English children after entry to primary school suggest that 30% and 27% of 5-7-year-old boys and girls respectively meet physical activity guidelines.^6^ In 2015, children aged 5-10 years spent 2.7 hours being sedentary on weekdays outside school and 3.6 hours at the weekend.^6^ When physical activity was assessed using device-based measures, approximately half of UK children aged 6-8 years met physical activity guidelines, boys were consistently more likely to engage in sufficient MVPA,^7,8^ and children tended to spend a large proportion of the day sedentary.^8,9^ Levels of physical activity then tend to decline over time, with decreases in MVPA and increases in sedentary time in both sexes between ages 6-8 years.^10^ Internationally, boys are more active than girls;^11,12^ this is particularly true in the after-school period (until 18:00) and at weekends for Australian 5-6-year-olds.^12^

The transition to school also impacts activity patterns over the course of the day and week, due to differing routines.^13^ In samples of primary school children, more activity is accumulated on weekdays compared with weekends, and children engage in more MVPA during school hours vs. outside of school.^13^ Yet, no studies to date have assessed how these changes are reflected in children’s activity behaviours during the transition to school (i.e. from preschool to formal schooling). Moreover, most studies have focussed on children’s average activity levels across measurement periods, which may mask differences seen when including daily variation,^14^ and provide only cross-sectional estimates of activity levels. Evidence is also equivocal about how a child’s weight status, socio-economic circumstances and siblings influence physical activity.^14^ Understanding these differences, and also variation across the day and week, allows intervention efforts to take account of when children may be less active and therefore open to efforts to increase activity.

Building on work conducted in the same sample at age 4 years,^14^ this paper describes levels and patterns of activity in a population-based sample of 6-year-old British children. Using children’ s daily activity levels, it examines activity patterns segmented across the day to determine how temporal and demographic factors influence activity levels and explores how physical activity levels change during the transition to formal schooling, between ages 4 and 6. We hypothesised that at age 6, children’s activity behaviours would differ by time of day and week in particular, given the added structure provided by the school day. Longitudinally, we theorised that children would spend more time sedentary at age 6 compared to age 4.

## Methods

### Study design and setting

The Southampton Women’s Survey (SWS) is a UK population-based prospective cohort study.^15^ The study recruited 12,583 non-pregnant women from General Practices in the Southampton area 1998-2002. Subsequent live singleton births (n=3,158) were assessed at specific ages to observe how children’s pre-natal development interacts with their postnatal growth, and how both affect risk factors for future chronic diseases.^15^ Between March 2006 and June 2009, a sub-study investigated physical activity, with all SWS children aged 4 years during this period (n=1,065) invited to participate.^16^ Those born after January 2000 were subsequently approached for a visit at age 6-7 years (hereafter “6 years”, between March 2007 – August 2012), and asked to wear an activity monitor (n=802). 308 children attended both study visits and provided valid physical activity data at both ages 4 and 6 years; 1003 children had valid data at one and/or other time point. The latter are included in the multi-level longitudinal analyses to provide a more complete sample, alongside sensitivity analyses on the former (see below). Parents (predominantly mothers) completed a questionnaire assessing sociodemographic and lifestyle information at both ages, and provided informed written consent for their child to participate. Ethical approval for SWS data collection at ages 4 and 6 was granted by the Southampton and South West Hampshire Local Research Ethics Committee.

### Physical Activity Assessment

As at age 4,^16^ at age 6, children were fitted with a combined heart rate and movement sensor (Actiheart, Cambridge Neurotechnology Ltd, UK) to measure free-living physical activity. The monitor was secured to the chest, and set to record at 60-second epochs to allow sufficient memory to record for 7 days. While the use of 60-second epochs may underestimate MVPA^17^ and overestimate LPA,^18^ the Actiheart has been shown to be valid in this age group.^19^ Participants were asked to wear the monitor continuously for seven days, including during sleep and water-based activities. Monitors were returned by secure post, along with the validated^20^ physical activity questionnaire.

### Outcome Measures

Only accelerometer data were used for these analyses, as non-individually calibrated heart-rate data explain little additional variation in estimates of free-living activity in similar aged children.^19^ Actiheart data were processed using Stata 14/SE.^21^ At both time points, data periods of 100 minutes or more with zero-activity counts were removed for all participants,^22^ as were days with <600 minutes of recording, with 10 hours of activity being the minimum cut-off to define a valid day.^23^ Children with ≥3 days of valid physical activity data at (age 4 and) age 6 were included in (longitudinal) analyses. Recordings between 23:00 and 06:00 were removed, as were those between 21:00 and 23:00 if they included more than 45 minutes of sedentary time, deemed to reflect time sleeping. This provides a conservative estimate of sleep time,^24^ whilst minimising over-estimation of sedentary time in the evenings.

For cross-sectional analyses at age 6 years, physical activity was derived as time spent (min/day) in two broad intensity categories using previously validated cutpoints: sedentary (SED: <20 counts/minute); and moderate-to-vigorous (MVPA: ≥460 counts/minute).^25^ Cutpoints were scaled using a conversion factor of 5 from validation work using the Actigraph accelerometer (Actigraph, Pensacola, FL, USA),^26^ as done previously in children of this age.^16^ As physical activity guidelines differ for children at ages 4 and 6, light activity was also used as an outcome for longitudinal analyses (LPA: >20-459 counts/minute).

### Exposure and confounding variables

A range of putative exploratory variables were examined in relation to children’s physical activity.^27,28^ The accelerometer output provided hour, time of day, weekday vs. weekend, and date (from which season was derived: winter December-February; spring March-May; summer June-August; autumn September-November). Each day was split into three periods: morning (07:00-09:00), school (09:00-15:00) and evening (15:00-23:00). Child’s sex and measured height and weight were recorded at ages 4 and 6 years,^29^ and used to calculate body mass index (BMI) (kg/m^2^) and sex/age-adjusted BMI z-score.^30^ Children were categorized as under-weight, normal or over-weight/obese using the International Obesity Task Force^31^ classifications.

The age mother left full-time education (≤16 years, 17-18 years, >18 years) was used as a proxy for socio-economic status, and maternal BMI was calculated from reported height and weight at ages 4 and 6 years, with WHO^32^ category classifications used for descriptive purposes. Similarly, data collected at ages 4 and 6 were used to describe the presence of siblings living in the cohort child’s home (younger siblings; older siblings; older and younger siblings). Where relevant, confounders comprised a measure of diet quality at age 6 (weekly fruit and vegetable consumption), a maternal physical activity score (derived using the RUPE index, found to have good reliability and validity in assigning scores to self-reported physical activity^33^) and derived child’s screen time from reported TV viewing (see Direct Acyclic Graphs (DAGs) in supplementary figures 1-7).

### Statistical analysis

Analyses were carried out using Stata/SE 14.^34^ Basic descriptive characteristics (e.g. child age, age mother left full-time education) at age 6 were calculated and compared with (a) the original SWS cohort (but not included here) and (b) those providing longitudinal data at ages 4 and 6.

### Cross-sectional analyses at age 6

Using children’s daily minutes spent sedentary or in MVPA, and these activity behaviours segmented across the day (morning, school, evening), as outcomes, two-level random intercept models were used to model associations between children’s daily activity and exposure variables. Hierarchical models allowed for variation across days (level 1) within children (level 2).^35^ Correlations between observations were accounted for by allowing the intercept to vary randomly between children (i.e. level 2). Exposures were child’s sex, child BMI category, siblings in the home at age 6, age mother left full-time education, time of the week (weekday vs. weekend) and season. Models were differentially (and minimally) adjusted for appropriate confounders and competing exposures, derived based on existing literature and using the Daggity software^36^ (see DAGs in supplementary figures 1-6). As there was no evidence of interaction effects by sex, data for the whole cohort (i.e. boys and girls together) are presented.

### Longitudinal analyses

For children who provided valid accelerometer data at ages 4 and/or 6 (n=1003, ‘combined’ sample), we examined change in activity behaviours/intensity over the two year period. Again, two-level random intercept models were fitted with child’s daily minutes sedentary, in LPA and in MVPA entered into the model as outcomes (level 1: day; level 2: child). An ‘age’ term (age 4 or 6) was also included to assess whether activity behaviours changed over time. Models were adjusted for sex, ethnicity, child screen time, maternal education, time of week and season (see DAG in supplementary figure 7). *Posthoc* interactions between ‘age’ and time of week/child’s sex based on *a priori* hypotheses, were assessed. We also limited the analysis to the 308 children who had valid physical activity data at both ages 4 and 6 (‘complete case’ sample). As this had little impact on the overall estimates, all children (n=1003) with valid data at one/both time points were retained to minimise bias for the posthoc analyses, but both the combined sample and complete case analyses are presented in Figure 1.

Sensitivity analyses were conducted to assess whether findings were influenced by a) excluding data collected in August (UK school summer holidays) for cross-sectional analyses only, and b) differing season of assessment between ages 4 and 6 (in 75% of children). As only small differences were observed in regression coefficients (i.e. difference in beta of <0.2 minutes) we present results from the original analyses.

## Results

712 children (89% of those fitted with monitors) had valid physical activity data at age 6 years, with a mean (SD) day-time wear of 14.0 (0.6) hours over 6.0 (1.3) valid days. Compared with the sample of SWS children who participated in any part of the age 6 follow up, those providing Actiheart data were slightly younger (6.6 (0.1) vs 6.7 (0.1)) and had mothers’ who left full time education slightly later (18.1 (0.1) vs. 17.9 (0.1)). The latter was also true when comparing children who provided Actiheart data at age 6 with the original SWS cohort. For the longitudinal analysis, there were no demographic differences between children providing data at one or two time points. Table 1 shows descriptive characteristics for included children (with average activity levels in Supplementary Table 1). At age 6, children were sedentary for mean 316.2 (68.2) minutes, engaged in 457.1 (65.4) minutes of LPA and 64.9 (25.7) minutes of MVPA. Just over half of the children (52.9%) met the current UK recommended guidelines of an average of ≥60 minutes of MVPA, with boys being more likely to meet physical activity guidelines than girls (62.5 vs 42.2%, *t-test* p<0.005).

### Cross-sectional analyses

At age 6, cross-sectional associations of children’s daily time spent sedentary and in MVPA showed that girls engaged in less MVPA than boys (MVPA: ß=-13.1 [95%C.I. -17.2,-9.1] minutes/day) (Tables 2, 3). Children engaged in more sedentary time and less MVPA at weekends (vs. weekdays); did more MVPA in spring (vs. winter); and those whose mothers left full time education after age 18 (vs. before 16 years) had lower levels of MVPA.

Multiple differential associations were identified for both outcomes when activity was segmented across the day (Tables 2, 3). Compared with boys, girls engaged in more sedentary time and less MVPA during school hours, with differences of a similar magnitude (i.e. SED: 7.1 [1.4,12.9]; MVPA: -8.1 [-10.4,-5.8] minutes/day). At the weekend (vs weekdays), children were more sedentary during mornings and evenings, and engaged in less MVPA during mornings/what would be school hours. In spring (vs. winter), children were less sedentary and engaged in more MVPA after 15:00 hours; in summer (vs. winter) children were more sedentary and did less MVPA before 09:00 but were less sedentary and engaged in more MVPA after 15:00. There were also differences by child’s weight status, the age mothers left full time education, and age of siblings living in the home. For the latter, those with younger siblings only were less sedentary in the mornings, and those with both younger and older siblings engaged in more MVPA in evenings.

### Longitudinal analyses

Longitudinally, at age 6, children became more sedentary (29.4 [24.6,34.2] minutes/day) and engaged in more MVPA (7.1 [5.2,9.1] minutes/day) but did less LPA (-43.0 [-47.5,-38.5] minutes/day) on average compared with when they were aged 4 (Figure 1, Supplementary Table 2). The estimates for the combined sample were slightly more conservative for SED and LPA than the complete case analysis, but both showed very similar patterns of change across the intensity distribution. *Posthoc* interaction analyses on the combined sample (n=1003) showed that the pattern of change was similar for boys and girls but the magnitude of change over time was lower for girls (Supplementary Table 3a). An age by time of week interaction indicated that the above pattern held for weekdays, and sedentary time and LPA at weekends, but there was no change in weekend MVPA between ages 4 and 6 (Supplementary Table 3b).

## Discussion

This is the first study to assess sedentary time and physical activity levels in 6-year-old British children segmented over the course of the day, with examination of changes in the levels and patterns of activity intensity during the transition to formal schooling. Cross-sectionally at age 6, boys were less sedentary and more active than girls; children engaged in more sedentary time and less MVPA at weekends (vs. weekdays); did more MVPA in spring (vs. winter); and children whose mothers left full time education after age 18 (vs. before 16 years) engaged in less MVPA. Subgroup differences were also identified when activity behaviours were segmented across the day. These time-specific observations provide useful information about when and where children may be more/less active, over and above indications provided by average daily physical activity. After entry into formal schooling, children engaged in less LPA but spent more time being sedentary and in MVPA, indicating that the relative intensity of children’s activity behaviours may change as they transition to formal education. Taken together, this information is useful for targeting focused interventions and resources towards periods where children may be less active. Although the transition to school appears to be a positive catalyst for MVPA, it is accompanied by larger increases in sedentary time, particularly for girls and at weekends, corroborating the wider trend of increased sedentary time as children age.^2^ Counteracting this gradual increase earlier in childhood may be one way to attenuate a potentially problematic behaviour, breaking the trend before it becomes habitual.

Similar to other samples of UK children,^7,8^ just under 50% of children here did not meet current UK activity guidelines, though boys were more likely to meet them than girls. Such differences in physical activity by child’s sex emerge in early life^28^ and remain as children progress through childhood into adulthood.^37^ Stratified analyses by time of day indicated girls may engage in less MVPA during the school day at age 6. Moreover, the magnitude of change in girls’ sedentary, LPA and MVPA was lower than in boys. It remains to be determined why this is the case. Change in girls’ activity behaviours may be smaller as they start from lower overall levels, or the type of activities young children choose or are offered may differ over time. This is particularly true of school-based PE lessons and break-time activities, where for example school uniforms for girls (i.e. skirts, dresses) are noted to be a major barrier to physical activity.^38^ Efforts to encourage higher intensity physical activity in primary schools for girls may therefore be warranted.

In this sample at age 4,^39^ as we hypothesized, differences in activity behaviours occur across the day, over and above those identified for children’s average daily activity behaviours. For example, children were more sedentary and engaged in less MVPA in the mornings at weekends (vs weekdays), likely reflecting increased active travel during this time on school days. In the mornings (07:00-09:00), children with younger siblings only were less sedentary, possibly a result of overall earlier wake-up times, or having someone to play with. All children engaged in less MVPA during 09:00 to 15:00 (school hours on weekdays), indicating targeted family-based interventions at the weekend may be warranted. Seasonally, in spring and summer (vs. winter), children were less sedentary and engaged in more MVPA after 15:00. Such variation in physical activity levels have been noted in similar-aged children;^40^ our work indicates that the after-school period during cooler months in the UK could be a key time for implementing interventions to ensure consistent levels of physical activity year-round. Regardless, focusing on children’s overall activity levels may mask instances during the day when children are more or less active. Considering how activity behaviours differ across the day, and not simply exploring factors associated with children’s average physical activity levels, can provide important contextual information about children’s activity behaviours.

Longitudinally, we demonstrated a shift in children’s activity intensity distribution over time. After the transition to school, they spent more time sedentary and in MVPA, but less time in LPA. Differences in the magnitude of change were noted by sex and time of the week. This likely reflects changing activity behaviours and differing routines as the children grew older, highlighting the possible importance of school for MVPA at age 6. UK physical activity guidelines at the time of measurement made a general recommendation for 4-years-olds to be active for 180 minutes per day, and for children aged 5 or over to engage in 60 minutes of MVPA.^41^ This may in part explain why MVPA increases at age 6, with educators and care-providers being more aware of the need for MVPA in older school-aged children. Current recommendations for both 4- and 6-year-olds now include 60 minutes of MVPA per day (on average);^4^ identifying exactly what drives this positive change in MVPA (of around ~7.5 minutes/day at age 6) could provide important information for preschool settings, allowing them to mirror opportunities for positive gains in MVPA.

Despite positive increases in MVPA, our findings support our hypothesis, and a general tendency, of increasing sedentary time across early-middle childhood in UK children. Cross-sectionally at age 6, children were sedentary for 38% of their day, or approximately 5.4 hours/day. This is less than in other UK samples of slightly older children, where at least half were sedentary for 6.4 hours or more,^8^ or spent 77.8% of their day sedentary.^9^ Another large UK study showed sedentary time increased by >75 minutes per week between ages 6 and 8.^10^ The population, measurement device used and wear/processing protocols likely contribute to differences between these population-based samples. We used Actiheart monitors, which are likely to capture children’s activity over the course of the whole day (they are not removed at bedtime or during water-based activities). Comparison across studies is therefore difficult, and greater consistency in measurement protocols is required for better surveillance between samples and over time.

Although the health benefits of LPA remain to be elucidated, the detrimental and beneficial effects of sedentary time and MVPA respectively continue to emerge for young children.^4^ The importance of activity across the whole intensity spectrum needs to be determined for younger children, as does the possibility that unfavourable impacts of increased sedentary time may be offset by similar increases in moderate or vigorous physical activity over time.

### Strengths and Limitations

This study is one of the first to describe device-measured activity behaviours during the transition to formal schooling, doing so in a large population-based sample of British 6-year-old children. Analysed cross-sectionally and longitudinally, the time-stamped data allowed description of patterns of physical activity across different times of day, providing a detailed overview of children’s activity behaviours, including potential substitution effects across the day at age 6. Although we considered a range of confounders, residual confounding cannot be ruled out. We divided the day into distinct time periods to capture before, during and after school time. While likely to be valid for the vast majority of children, some of the morning and evening time may also include school time, and will definitely include travel to/from school. Although we do not have contextual information about activities associated with physical activity, this must also be born in mind. There were no demographic differences between those who provided data at one or more (combined) vs. both time points (complete case). Findings from the longitudinal analysis with the combined and complete case samples were very similar, with the combined analysis providing a more conservative estimate of SED and LPA; this suggests that these data are likely to be representative of those participating at age 6. However, those participating at age 6 had more educated mothers than the original SWS sample, and those providing Actiheart data at age 6 were slightly younger and had more educated mothers than children who participated in any part of the age 6 follow up. Limited available evidence suggests that children’s physical activity at this age is not influenced by socio-economic circumstances,^42^ with age differences between those who did and did not provide data here also being minimal (0.1 years). As with any cohort, we cannot however rule out attrition leading to those who remain in the study not being truly representative of the original cohort. Fewer children in this sample were overweight or obese compared with the national average, and participants were predominately white British. Although this is broadly in keeping with the Southampton region (where census data indicated ~82% were White British),^43^ it suggests that the sample may not be representative of the British population of preschool/school-aged children as a whole.

## Conclusion

Just over 50% of this sample of 6-year-old British children met activity guidelines, with activity patterns varying across the day, week and by demographic factors. At age 6, children were more sedentary but also engaged in more MVPA each day compared to when they were aged 4. Together, this highlights the continued need to promote positive activity behaviours in young school-aged children, but also to consider both ends of the activity intensity distribution, as positive and negative changes may occur simultaneously. Demographic and temporal factors, and a focus on intensity, should be considered when developing interventions to encourage positive activity behaviours in young children, allowing efforts to be targeted to achieve the largest incremental positive change in outcomes.

## Supplementary Material

S1.

Supplementary Material

## Figures and Tables

**Figure 1:** Longitudinal analysis exploring change in activity behaviours between age 4 and age 6, with combined (n=1003) and complete case (n=308) samples

**Table 1:** Characteristics of included children with valid Actiheart data

Variable	Cross-sectional (n=712)	Longitudinal^^^ (n=1003)
Boys (n (%))	368 (51.7)	524 (52.2)
Age (years)	6.6 (0.1)	6.6 (0.1)
Ethnicity (n (%) non-white)	37 (5.2)	51 (5.1)
Birthweight (g)	3472.7 (521.6)	3480.5 (509.6)
Height* (cm)	120.3 (5.3)	-
Weight* (kg)	23.2 (3.7)	-
BMI* (kg/m^2^)	16.0 (1.4)	-
BMI z-score*	0.16 (0.97)	-
Weight status* (IOTF classification) (n (%))		
Thin	38 (5.4)	-
Normal	565 (80.4)	-
Overweight/Obese	102 (14.5)	-
Age Mother left Education (n (%))		
≤16 years	249 (35.0)	350 (34.9)
17-18 years	259 (36.4)	356 (35.5)
≥18 years	2203 (28.6)	297 (29.6)
Maternal parity at child’s birth		
0	350 (49.2)	511 (51.0)
1	263 (36.9)	360 (35.9)
2/+	99 (13.9)	122 (13.1)
Siblings at home* (n (%))		
None	164 (21.6)	-
Older only	262 (34.5)	-
Younger only	247 (32.5)	-
Older and younger	87 (11.5)	-

All values are mean (SD) unless stated otherwise; SD: standard deviation

^ Children with accelerometry at one or both time points

* indicates measured at age 6; BMI: Body Mass Index

IOTF: International Obesity Task Force.

**Table 2:** Associations between temporal and demographic factors and children’s sedentary behaviour at age 6 (n=712)

	Daily total	Morning 07:00-09:00	School hours 09:00-15:00	Evening 15:00-23:00
		B [95% C.I.] (Minutes sedentary/day)		
Sex^1^ (ref: boys)	2.2	-0.94	**7.1**	-3.1
	[-9.0,13.4]	[-3.6,18]	**[1.4,12.9]**	[-9.1,2.9]
BMI^2^ (ref: normal)				
Underweight	-2.0	1.8	2.2	-5.8
	[-30.0,25.9]	[-5.4,9.0]	[-12.2,16.44]	[-21.3,9.7]
Overweight/obese	-1.7	-1.3	-6.2	3.7
	[-21.4,18.0]	[-6.2,3.7]	[-16.3,3.8]	[-7.0,14.5]
Age mother left education^3^ (ref <16 years)				
17-18 years	-0.76	**-3.0**	2.4	-0.33
	[-12.5,11.0]	**[-6.0,-0.06]**	[-3.9,8.6]	[-6.6,5. 9]
>18 years	2.2	**-4.8**	2.7	2.2
	[-10.2,14.6]	**[-7.9,-1.6]**	[-3.9,9.3]	[-4.4,8.7]
Siblings^4^ (ref: none)				
Older only	-5.4	0.51	-0.38	-5.5
	[-18.7,7.9]	[-2.8,3.8]	[-7.4,6. 7]	[-12.5,1.5]
Younger only	-10.0	**-4.6**	-6.8	1.7
	[-23.5,3.6]	**[-8.0,-1.2]**	[-14.1,0.43]	[-5.5,8.9]
Older and younger	-15.5	-3.8	-4.3	-8.9
	[-33.4,2.4]	[-8.2,0.65]	[-13.8,5.2]	[-18.3,0.53]
Time of week^5^ (ref: weekday)	**25.3**	**18.6**	-2.1	**11.6**
	**[18.2,32.3]**	**[16.6,20.6]**	[-5.8,17]	**[6.9,16.4]**
Season^6^ (ref: Winter)				
Spring	-12.5	1.8	-0.98	**-10.2**
	[-28.0,3.0]	[-2.0,5.5]	[-9.0,7.0]	**[-18.5,-1.9]**
Summer	-2.4	**8.9**	1.2	**-10.2**
	[-18.5,13.7]	**[5.1,12.8]**	[-7.0,9.5]	**[-18.8,-1.7]**
Autumn	1.7	-0.64	5.5	1.4
	[-14. 6,17.9]	[-4.5,3.3]	[-2.9,13.8]	[-7.2,9.9]

95% confidence intervals in brackets; bold text indicates that confidence intervals do not overlap 0

1 Sex adjusted for ethnicity, diet, maternal education, maternal BMI, maternal physical activity, siblings, time of the week and season

2 BMI adjusted for sex, ethnicity, child screen time, maternal education, maternal BMI, and time of the week

3 Maternal education adjusted for sex, ethnicity, time of week and season

4 Siblings adjusted for ethnicity, maternal education, time of week and season

5 Time of week adjusted for sex, ethnicity, diet, child BMI, child screen time, maternal education, maternal BMI, maternal physical activity, siblings and season

6 Season adjusted for sex, ethnicity, maternal education, maternal BMI, siblings and time of week.

**Table 3:** Associations between temporal and demographic factors and children’s MVPA at age 6 (n=712)

	Daily total	Morning 07:00-09:00	School hours 09:00-15:00	Evening 15:00-23:00
		B [95% C.I.] (Minutes in MVPA/day)		
Sex^1^ (ref: boys)	**-13.1**	-0.50	**-8.1**	**-4.8**
	**[-17.2,-9.1]**	[-1.2,0.17]	**[-10.4,-5.8]**	**[-7.0,-2.5]**
BMI^2^ (ref: normal) underweight	-1.1	-1.2	-2.2	**5.9**
	[-11.3,9.1]	[-3.0,0.54]	[-8.0,3.7]	**[0.13,11.7]**
Overweight/obese	-5.3	0.61	-1.5	**-4.5**
	[-12.5,1.9]	[-0.62,1.8]	[-5.6,2.6]	**[-8.5,-0.47]**
Age mother left education^3^ (ref <16 years)				
17-18 years	0.88	0.26	-0.02	-0.04
	[-3.3,5.1]	[-0.43,0.94]	[-2.4,2.4]	[-2.4,2.3]
>18 years	-4.8	0.11	-1.9	-2.9
Siblings^4^ (ref: none)	[-9.2,-0.32]	[-0.62,0.85]	[-4.5,0.63]	[-5.3,-0.37]
Older only	1.9	-0.22	-0.03	2.0
	[-3.0,6.9]	[-0.98,0.59]	-2.9, 2.8]	[-0.63,4.7]
Younger only	-1.2	0.35	-0.13	-1.6
	[-6.3,3.8]	[-0.46,1.2]	[-3.1,2.8]	[-4.4,1.1]
Older and younger	6.1	0.18	1.2	**4.0**
	[-0.52,12.8]	[-0.87,1.2]	[-2.6,5.1]	**[0.42,7.6]**
Time of week^5^ (ref: weekday)	**-14.5**	**-4.3**	**-9.2**	-1.1
	**[-17.3,-11.7]**	**[-4.8,-3.8]**	**[-11.0,-7.4]**	[-2.9,0.62]
Season^6^ (ref: Winter)				
Spring	**8.7**	-0.44	1.7	**7.6**
	**[3.2,14.2]**	[-1.4,0.48]	[-15,4.9]	**[4.6,10.7]**
Summer	2.1	**-1.3**	-2.5	**6.0**
	[-3.6,7.9]	**[-2.3,-0.39]**	[-5.8,0.81]	**[2.8,9.1]**
Autumn	-0.77	0.11	-1.4	0.84
	[-6.6,5.0]	[-0.85,1.1]	[-4.7,2.0]	[-2.3,4.0]

95% confidence intervals in brackets; bold text indicates that confidence intervals do not overlap 0

1 Sex adjusted for ethnicity, diet, maternal education, maternal BMI, maternal physical activity, siblings, time of the week and season

2 BMI adjusted for sex, ethnicity, child screen time, maternal education, maternal BMI, and time of the week

3 Maternal education adjusted for sex, ethnicity, time of week and season

4 Siblings adjusted for ethnicity, maternal education, time of week and season

5 Time of week adjusted for sex, ethnicity, diet, child BMI, child screen time, maternal education, maternal BMI, maternal physical activity, siblings and season

6 Season adjusted for sex, ethnicity, maternal education, maternal BMI, siblings and time of week.

## References

[1] Janssen I, Leblanc AG (2010). Systematic review of the health benefits of physical activity and fitness in school-aged children and youth. Int J Behav Nutr Phys Act.

[2] Nader PR, Bradley RH, Houts RM, McRitchie SL, O’Brien M (2008). Moderate-to-vigorous physical activity from ages 9 to 15 years. J Am Med Assoc.

[3] NHS Digital (2019). Statistics on Obesity, Physical Activity and Diet.

[4] Department of Health and Social Care (2019). UK Chief Medical Officers ‘ Physical Activity Guidelines.

[5] World Health Organisation (2020). WHO Guidelines on Physical Activity and Sedentary Behavior.

[6] NHS Digital (2015). Health Survey for England.

[7] Jago R, Sebire SJ, Wood L (2014). Associations between objectively assessed child and parental physical activity: A cross-sectional study of families with 5-6 year old children. BMC Public Health.

[8] Griffiths LJ, Cortina-Borja M, Sera F (2013). How active are our children? Findings from the Millennium Cohort Study. BMJ Open.

[9] King AC, Parkinson KN, Adamson AJ (2011). Correlates of objectively measured physical activity and sedentary behaviour in English children. Eur J Public Health.

[10] Jago R, Solomon-Moore E, Macdonald-Wallis C, Sebire SJ, Thompson JL, Lawlor DA (2017). Change in children’s physical activity and sedentary time between Year 1 and Year 4 of primary school in the B-PROACT1V cohort. Int J Behav Nutr Phys Act.

[11] Gronholt Olesen L, Lund Kristensen P, Korsholm L, Boye Koch A, Froberg K (2015). Correlates of objectively measured physical activity in 5-6-year-old preschool children.

[12] Cleland V, Crawford D, Baur LA, Hume C, Timperio A, Salmon J (2008). A prospective examination of children’s time spent outdoors, objectively measured physical activity and overweight. Int J Obes.

[13] Brooke HL, Corder K, Atkin AJ, van Sluijs EMF (2014). A Systematic Literature Review with Meta-Analyses of Within- and Between-Day Differences in Objectively Measured Physical Activity in School-Aged Children. Sport Med.

[14] Hesketh K, O’Malley C, Mazarello Paes V Determinants of Change in Physical Activity in Children 0-6 years of age: A Systematic Review of Quantitative Literature. Sport Medincine.

[15] Inskip HM, Godfrey KM, Robinson SM, Law CM, Barker DJ, Cooper C (2006). Cohort profile: The Southampton Women’s Survey. Int J Epidemiol.

[16] Hesketh KR, Goodfellow L, Ekelund U (2014). Activity Levels in Mothers and Their Preschool Children. Pediatrics.

[17] Vale S, Santos R, Silva P, Soares-Miranda L, Mota J (2009). Preschool children physical activity measurement: importance of epoch length choice. Pediatr Exerc Sci.

[18] Reilly JJ, Penpraze V, Hislop J, Davies G, Grant S, Paton JY (2008). Objective measurement of physical activity and sedentary behaviour: review with new data. Arch Dis Child.

[19] Corder K (2007). Physical Activity Measurement in Young People. MRC Epidemiol Unit.

[20] McMinn AM, van Sluijs EM, Harvey NC (2009). Validation of a maternal questionnaire on correlates of physical activity in preschool children. Int J Behav Nutr Phys Act.

[21] StataCorp LP (2013). STATA 13/SE.

[22] Choi L, Liu Z, Matthews CE, Buchowski MS (2011). Validation of accelerometer wear and nonwear time classification algorithm. Med Sci Sport Exerc.

[23] Beets MW, Bornstein D, Dowda M, Pate RR (2011). Compliance with national guidelines for physical activity in U.S. preschoolers: measurement and interpretation. Pediatrics.

[24] Acebo C, Sadeh A, Seifer R, Tzischinsky OO, Hafer A, Carskadon MA (2005). Sleep/wake patterns derived from activity monitoring and maternal report for healthy 1- to 5-year-old children. Sleep.

[25] Evenson KR, Catellier DJ, Gill K, Ondrak KS, McMurray RG (2008). Calibration of two objective measures of physical activity for children. J Sports Sci.

[26] Ridgway CL, Brage S, Sharp SJ (2011). Does birth weight influence physical activity in youth? A combined analysis of four studies using objectively measured physical activity. PLoS One.

[27] Hesketh McMinn AM, Sharp S, Ekelund U, Collings PJ, Harvey N, Inskip H, Godfrey K, Cooper C, Van Sluijs EKR Patterns of objectively measured physical activity in 4-year-old British children. Int J Behav Nutr Phys Act.

[28] Hinkley T, Crawford D, Salmon J, Okely AD, Hesketh K (2008). Preschool children and physical activity: a review of correlates. Am J Prev Med.

[29] Harvey NC, Mahon PA, Robinson SM (2010). Different indices of fetal growth predict bone size and volumetric density at 4 years of age. J Bone Miner Res.

[30] Cole TJ (1990). The LMS method for constructing normalized growth standards. Eur J Clin Nutr.

[31] Cole TJ, Bellizzi MC, Flegal KM, Dietz WH (2000). Establishing a standard definition for child overweight and obesity worldwide: international survey. Br Med J.

[32] World Health Organisation BMI Classification.

[33] Wareham NJ, Jakes RW, Rennie KL (2003). Validity and repeatability of a simple index derived from the short physical activity questionnaire used in the European Prospective Investigation into Cancer and Nutrition (EPIC) study. Public Health Nutr.

[34] StataCorp LP (2012). Stata/SE 12 for Windows.

[35] Goldstein H (2010). Multilevel Statistical Models.

[36] Textor J, Hardt J (2011). DAGitty: A graphical tool for analyzing causal diagrams. Epidemiology.

[37] Trost SG, Owen N, Bauman AE, Sallis JF, Brown W (2002). Correlates of adults’ participation in physical activity: review and update. Med Sci Sport Exerc.

[38] Allender S, Cowburn G, Foster C (2006). Understanding participation in sport and physical activity among children and adults: a review of qualitative studies. Heal Educ Res.

[39] Hesketh K, McMinn A, Ekelund U (2014). Objectively measured physical activity in four-year-old British children: A cross-sectional analysis of activity patterns segmented across the day. Int J Behav Nutr Phys Act.

[40] Atkin AJ, Sharp SJ, Harrison F, Brage SS, Van Sluijs EMF (2016). Seasonal Variation in Children’s Physical Activity and Sedentary Time. Med Sci Sports Exerc.

[41] Department of Health (2011). Start Active, Stay Active: A Report on Physical Activity from the Four Home Countries’ Chief Medical Officers.

[42] Hinkley T, Salmon J, Okely AD, Hesketh K, Crawford D (2012). Correlates of preschool children’s physical activity. Am J Prev Med.

[43] Southampton City Council and the Office for National Statistics (2011). Census Ethnicity and Religion.

