# Supplementary material for: Activity behaviours in British 6-year-olds: cross-sectional associations and longitudinal change during the school transition
Source: J Phys Act Health. Author manuscript; Available in PMC 2022 Sep 22. (PMC7613624; doi:10.1123/jpah.2021-0718)
Supplement: S1. [file EMS153636-supplement-S1_.docx]

Supplementary Figures

**Key**

DAG – Direct Acyclic Graph (derived using Daggity software)


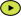
 exposure


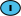
 outcome


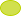
 ancestor of exposure


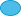
 ancestor of outcome


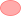
 ancestor of exposure *and* outcome


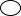
 adjusted variable


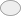
 unobserved (latent)


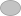
 other variable


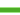
 causal path


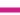
 biasing path

Figure 1 – DAG used for analyses assessing the cross-sectional association between child sex and children’s activity behaviours


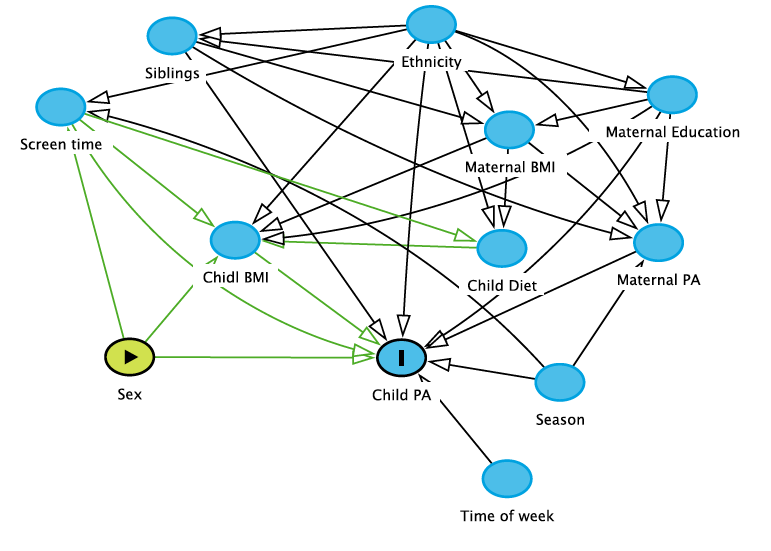


*Adjusted for ethnicity, diet, maternal education, maternal BMI, maternal physical activity, siblings, time of the week and season*

Figure 2 - DAG used for analyses assessing the cross-sectional association between child BMI and children’s activity behaviours


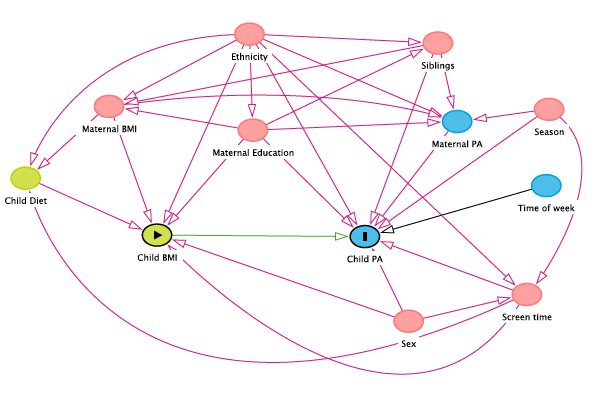


*Adjusted for sex, ethnicity, child screen time, maternal education, maternal BMI, and time of the week*

Figure 3 - DAG used for analyses assessing the cross-sectional association between age mother left education and children’s activity behaviours


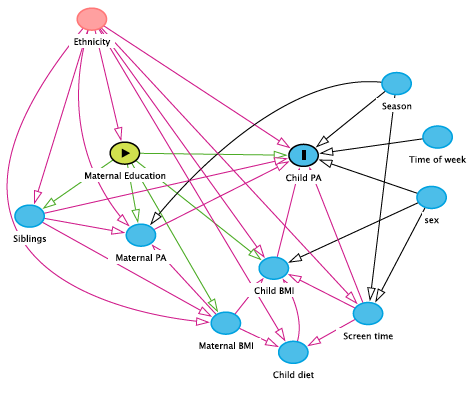


*Adjusted for sex, ethnicity, time of week and season*

Figure 4 - DAG used for analyses assessing the cross-sectional association between siblings in the home and children’s activity behaviours


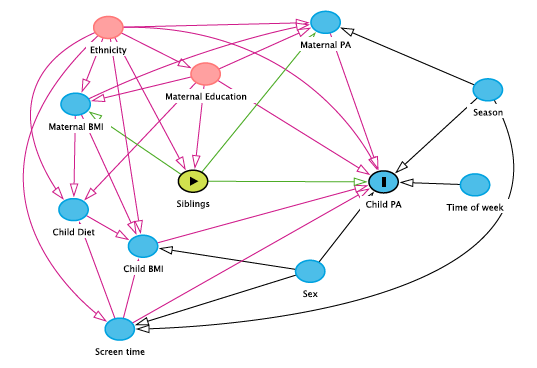


*Adjusted for ethnicity, maternal education, time of week and season*

Figure 5 - DAG used for analyses assessing the cross-sectional association between time of the week and children’s activity behaviours


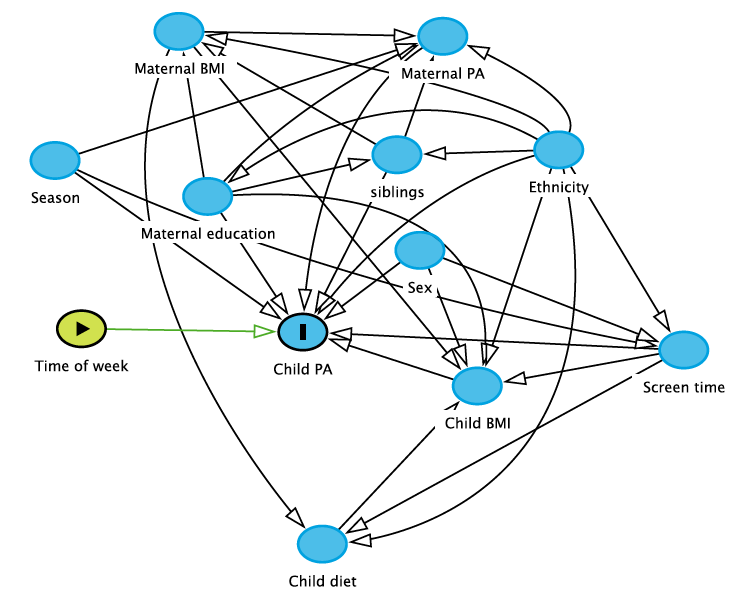


*Adjusted for sex, ethnicity, diet, child BMI, child screen time, maternal education, maternal BMI, maternal physical activity, siblings and season*

Figure 6 - DAG used for analyses assessing the cross-sectional association between season and children’s activity behaviours


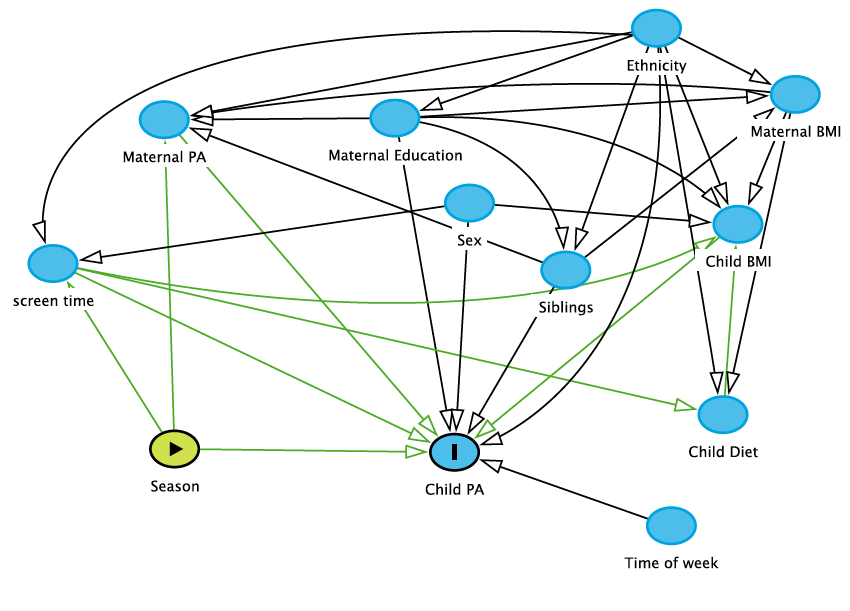


*Adjusted for sex, ethnicity, maternal education, maternal BMI, siblings and time of week*

Figure 7 - DAG used for analyses assessing the longitudinal association between child age and children’s activity behaviours


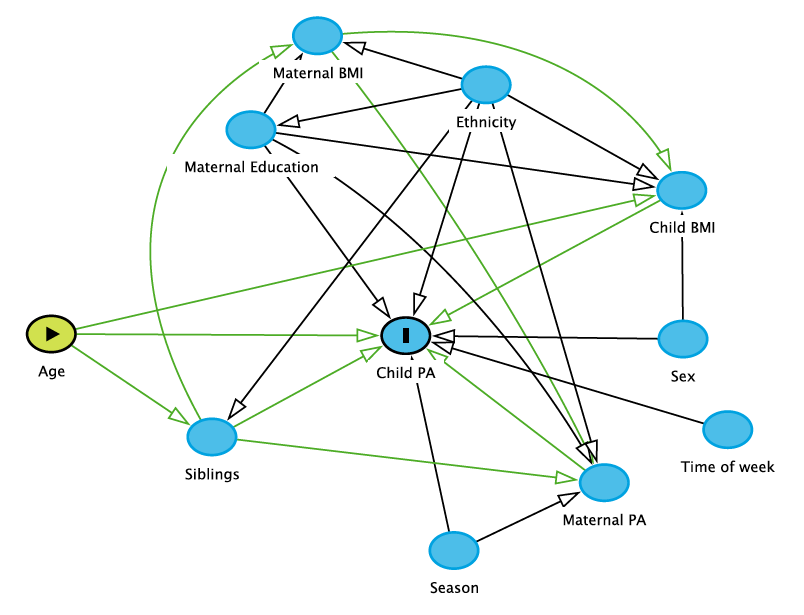


*Analyses adjusted for sex, ethnicity, maternal education, time of week and season*
