# Supplementary material for: Activity behaviours in British 6-year-olds: cross-sectional associations and longitudinal change during the school transition
Source: J Phys Act Health. Author manuscript; Available in PMC 2022 Sep 22. (PMC7613624; doi:10.1123/jpah.2021-0718)
Supplement: Supplementary Material [file EMS153636-supplement-Supplementary_Material.doc]

Supplementary Table 1: Average time children spent in each physical activity intensity at age 6

|  | Cross-sectional  Age 6 | | | | |
| --- | --- | --- | --- | --- | --- |
|  | Daily total | % time | Morning  07:00-09:00 | School  09:00-15:00 | Evening  15:00-23:00 |
| **Valid days** | **6.0 (1.3)** |  |  |  |  |
| Boys | 6.0 (1.3) |  |  |  |  |
| Girls | 6.0 (1.3) |  |  |  |  |
| **Valid hours** | **14.0 (0.6)** |  | **2.1 (0.4)** | **5.9 (0.4)** | **6.0 (1.0)** |
| Boys | 14.0 (0.6) |  |  |  |  |
| Girls | 13.9 (0.6) |  |  |  |  |
| Time (minutes/day) |  |  |  |  |  |
| **Sedentary** | **316.2 (68.2)** | **38** | **64.8 (17.4)** | **109.1 (51.7)** | **142.8 (58.9)** |
| Boys | 314.2 (68.4) | 37 | 64.5 (17.4) | 106.9 (33.0) | 144.3 (36.2) |
| Girls | 318.3 (68.0) | 38 | 65.3 (17.6) | 112.7 (35.2) | 141.4 (32.7) |
| **Light PA** | **457.1 (65.4)** | **55** | **57.1 (21.3)** | **204.0 (55.6)** | **185.6 (63.7)** |
| Boys | 454.0 (66.9) | 54 | 58.1 (21.3) | 202.7 (32.0) | 182.2 (41.6) |
| Girls | 460.5 (63.7) | 55 | 55.3 (21.6) | 205.4 (34.0) | 188.7 (38.0) |
| **Moderate-vigorous PA** | **64.9 (25.7)** | **8** | **5.0 (4.2)** | **33.1 (23.3)** | **24.6 (22.3)** |
| Boys | 71.5 (27.1) | 9 | 4.9 (4.2) | 36.7 (14.7) | 26.9 (14.5) |
| Girls | 57.8 (22.2) | 7 | 4.4 (3.6) | 28.4 (12.1) | 22.6 (11.5) |

PA: Physical Activity; All values mean(standard deviation) unless stated.

Supplementary Table 2: Longitudinal analysis exploring change in activity behaviours between age 4 and age 6, in combined (n=1003) and complete case (n=308) samples

|  | SED | LPA | MVPA |
| --- | --- | --- | --- |
|  | B (95% Confidence Intervals)  Change in minutes/day between age 4 and 6 | | |
| Combined sample  n=1003 | 29.4 (24.6, 34.2) | -43.0 (-47.5, -38.4) | 7.1 (5.2, 9.1) |
| Complete case  n=308 | 32.3 (26.6, 38.0) | -46.1 (-51.4, -40.8) | 6.2 (3.7, 8.7) |

Analyses adjusted for sex, ethnicity, maternal education, time of week and season.

**Supplementary Table 3a: Post hoc longitudinal analyses examining change in activity behaviours between age 4 and age 6 for boys and girls (n=1003)**

| Boys (n=524) | SED | LPA | MVPA |
| --- | --- | --- | --- |
|  |  | B [95% C.I.]  (Minutes /day) |  |
| Minutes/ day (constant) | 286.6 (273.7, 299.6) | 493.7 (481.2, 506.1) | 60.4 (55.4, 65.4) |
|  |  |  |  |
| Age 6 (ref Age 4) | **31.8 (25.2, 41.7)** | **-50.0 (-56.7, -43.4)** | **11.1 (8.3, 13.9)** |

| Girls (n=479) | SED | LPA | MVPA |
| --- | --- | --- | --- |
|  |  | β [95% C.I.]  (Minutes /day) |  |
| Minutes/ day (constant) | 286.8 (273.6, 299.9) | 502.2 (489.9, 514.6) | 48.0 (42.8, 53.3) |
|  |  |  |  |
| Age 6 (ref Age 4) | **26.7 (20.2, 33.3)** | **-35.6 (-41.9, -29.5)** | **3.4 (0.6, 6.2)** |

95% confidence intervals in brackets; bold text indicates that confidence intervals do not overlap 0; analyses adjusted for sex, ethnicity, maternal education, time of week and season.

**Supplementary Table 3b: Post hoc longitudinal analyses examining change in activity behaviours between age 4 and age 6 on weekday and weekend days (n=1003)**

| Weekday (n=1003) | SED | LPA | MVPA |
| --- | --- | --- | --- |
|  |  | B [95% C.I.]  (Minutes /day) |  |
| Minutes/ day (constant) | 281.2 (254.9, 307.5) | 506.8 (483.0, 530.5) | 53.1 (44.2, 62.0) |
|  |  |  |  |
| Age 6 (ref Age 4) | **24.8 (19.5, 31.2)** | **-40.4 (-45.5, -35.3)** | **11.5 (9.3, 13.6)** |

| Weekend (n=896) | SED | LPA | MVPA |
| --- | --- | --- | --- |
|  |  | β [95% C.I.]  (Minutes /day) |  |
| Minutes/ day (constant) | 290.7 (259.8, 321.7) | 500.4 (471.7, 529.0) | 52.2 (40.1, 64.3) |
|  |  |  |  |
| Age 6 (ref Age 4) | **38.9 (30.4, 47.5)** | **-46.6 (-54.5, -38.6)** | -3.0 (-6.5, 0.5) |

95% confidence intervals in brackets; bold text indicates that confidence intervals do not overlap 0; analyses adjusted for sex, ethnicity, maternal education, time of week and season.

Figure 1: Longitudinal analysis exploring change in activity behaviours between age 4 and age 6, with combined (n=1003) and complete case (n=308) samples

Supplementary Figure 1 – DAG used for analyses assessing the cross-sectional association between child sex and children’s activity behaviours

Supplementary Figure 2 - DAG used for analyses assessing the cross-sectional association between child BMI and children’s activity behaviours

Supplementary Figure 3 - DAG used for analyses assessing the cross-sectional association between age mother left education and children’s activity behaviours

Supplementary Figure 4 - DAG used for analyses assessing the cross-sectional association between siblings in the home and children’s activity behaviours

Supplementary Figure 5 - DAG used for analyses assessing the cross-sectional association between time of the week and children’s activity behaviours

Supplementary Figure 6 - DAG used for analyses assessing the cross-sectional association between season and children’s activity behaviours

Supplementary Figure 7 - DAG used for analyses assessing the longitudinal association between child age and children’s activity behaviours

Supplementary Figures

KEY:


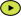
 exposure


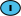
 outcome


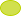
 ancestor of exposure


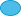
 ancestor of outcome


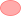
 ancestor of exposure *and* outcome


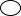
 adjusted variable


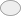
 unobserved (latent)


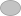
 other variable


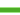
 causal path


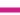
 biasing path
